# Use of Spatial Analysis to Test Hypotheses on Plant Recruitment in a Hyper-Arid Ecosystem

**DOI:** 10.1371/journal.pone.0091184

**Published:** 2014-03-10

**Authors:** Jan J. Quets, Stijn Temmerman, Magdy I. El-Bana, Saud L. Al-Rowaily, Abdulaziz M. Assaeed, Ivan Nijs

**Affiliations:** 1 Plant and Vegetation Ecology (PLECO), Department of Biology, University of Antwerp, Wilrijk, Belgium; 2 Ecosystem Management, Department of Biology, University of Antwerp, Wilrijk, Belgium; 3 Department of Botany, Faculty of Science, Port Said University, Port Said, Egypt; 4 Department of Plant Production, College of Agriculture, King Saud University, Riyadh, Saudi Arabia; DOE Pacific Northwest National Laboratory, United States of America

## Abstract

Mounds originating from wind-blown sediment accumulation beneath vegetation (nebkhas) often indicate land degradation in dry areas. Thus far, most nebkha research has focused on individual plants. Here, we aimed to explore population-scale processes (up to scales of about 100 m) that might explain an observed nebkha landscape pattern. We mapped the *Rhazya stricta* Decne. population in a 3 ha study site in a hyper-arid region of Saudi Arabia. We compared the spatial patterns of five different cohorts (age classes) of observed nebkha host plants to those expected under several hypothesized drivers of recruitment and intraspecific interaction. We found that all *R. stricta* cohorts had a limited fractional vegetation cover and established in large-scale clusters. This clustering weakened with cohort age, possibly indicating merging of neighboring vegetation patches. Different cohort clusters did not spatially overlap in most cases, indicating that recruitment patterns changed position over time. Strong indications were found that the main drivers underlying *R. stricta* spatial configurations were allogenic (i.e. not driven by vegetation) and dynamic. Most likely these drivers were aeolian-driven sand movement or human disturbance which forced offspring recruitment in spatially dynamic clusters. Competition and facilitation were likely active on the field site too, but apparently had a limited effect on the overall landscape structure.

## Introduction

Some desert-plant species are tolerant to abrasion and burial by sand [1]. When fine wind-borne sediment, organic matter, and litter are deposited around such plants, nutrient rich nebkhas (phytogenic mounds) are formed [2], [3]. The rate of rainfall infiltration through the soil is often higher under shrub canopies than in the surrounding bare soil [4], further enhancing the fertile-island effect of nebkhas [5]. Nebkhas occur naturally in sandy deserts [6], but they may also emerge in grasslands when vegetation cover is decreased by human disturbance or prolonged drought [7], [8], [9]. The presence of nebkhas may thus be an indicator of land degradation. However, because nebkhas always increase the aerodynamic roughness of the surface with respect to bare soil, they decrease wind speed close to the surface; this reduces wind transport of soil and acts as a restraint on desert expansion [1], [10]. In the light of ongoing desertification [11], nebkha studies are thus highly relevant.

Nebkhas typically occur isolated from each other and thus display patchiness at a landscape scale. Such vegetation patchiness is a widespread phenomenon [12], [13], [14], [15], [16], [17]. Theories explaining patchiness of vegetation are mostly based on recruitment limitation, either in the form of seed limitation (SL) [18] or habitat patchiness (HP) [19], [20], [21]. SL can arise when the union of all seed shadows (a seed shadow is the spatial distribution of dispersed seeds around their source [22]) in the landscape does not cover all study site locations, hereby creating vegetation-free landscape areas [18] (distance SL). However, even when seeds are locally present, the local seed density might be too low to locally produce full vegetation cover, while adding more seeds would locally increase vegetation cover [23] (density SL). Recruitment limitation due to HP might originate from vegetation-independent exogenous factors (allogenic drivers), or from the plants themselves due to competition or facilitation (autogenic drivers). Allogenic HP may, for example, arise from small-scale topographic depressions which redistribute precipitation water and therefore create small-scale habitat patches with higher than average soil moisture content, from large-scale immobile soil patches with characteristics discouraging seedling establishment (e.g. patches of highly compacted soil or poor nutrients), or due to recently deposited (or deflated) large-scale sheets of loose sand which suppress emergence and establishment of seedlings, even in pre-existing suitable habitats [2], [24], [25], [26], [27]. Autogenic HP can occur when plants compete with [17], [28], [29] or facilitate neighboring plants [20], [30]. Such autogenic conditions are mostly found close to the seed source, and have the most impact on neighboring young (i.e. vulnerable) individuals. Thus, both competition and facilitation may generate habitat patches for offspring establishment, and when they do, the spatial distribution of these habitat patches should spatially correlate with that of the vegetation imposing the competition (causing a negative correlation) or facilitation (causing a positive correlation). In some cases, autogenic processes are the only cause of vegetation patchiness, for instance in the case of self-organized vegetation patterns which arise from strong scale-dependent feedbacks (i.e. short-range positive autogenic effects combined with long-range negative autogenic effects). Self-organized vegetation patterns are typically highly regularly distributed in space [31].

Different ecological processes may give rise to distinct spatial vegetation patterns. In turn, such processes might be strongly suggested by observed vegetation patterns [32]. An effective way to indicate processes underlying real-world vegetation patterns is to *a priori* hypothesize a set of possible relevant processes which could produce specific vegetation patterns, and then to compare the observed vegetation pattern with the vegetation patterns expected to arise from each proposed process, by using refined spatial statistical techniques [32]. This approach has been used for disentangling ecological processes in various ecosystems [33], [34], [35], but rarely in nebkha landscapes [17], [36]. Here, we applied this approach to a vegetation pattern of *Rhazya stricta* Decne. (a widespread nebkha-forming shrub) in a hyper-arid Saudi Arabian field site. We evaluated hypotheses on recruitment and establishment of *R. stricta* nebkhas, using modified point pattern analyses on remotely sensed *R. stricta* patterns.

## Methods

### Study site

The study site (25.510° N, 46.002° E; 631 m altitude; 2.86 ha) lies 120 km northwest of Riyadh city (Saudi Arabia), and approximates a rectangle of 125 m×250 m, with the long side aligned SSW-NNE. The site is within a larger area under the control of the Department of Natural Resources of the Saudi Arabian Ministry of Agriculture. However, no official permission was needed to carry out our fieldwork. There were no endangered or protected species within the field site or in its surroundings. Riyadh annual precipitation and pan evaporation amount to 83 and 2816 mm respectively [37], which reflects a hyper-arid climate [38]. Precipitation falls between October and May, and is highly variable both in space and time. Mean daily temperature is lowest (14.2°C) in January and peaks (33.7°C) in July [37]. The study site's surface soil consisted of an upper loose sandy surface layer (with a variable thickness of up to 0.50 m) without a surface crust, and bedded on top of a hard cemented floor. Roots of established vegetation were able to penetrate the cemented soil layer, despite of its consolidation. Textures of both layers were sandy clay loam (USDA texture triangle). CaCO_3_ levels were significantly higher in the cemented soil as compared to the loose sandy layer on top of it (5.5 wt% versus 2.5 wt%; t-test; p<0.001), indicating that the cementation was associated with CaCO_3_, but other causes of cementation cannot be excluded.

The site is dominated by *R. stricta* (Apocynaceae), a regionally common unpalatable shrub. In most areas of the Arabian Peninsula, palatable species are heavily overgrazed by camels [39], [40], [41], which gives an advantage to the unpalatable *R. stricta* population. After the unbranched stage of *R. stricta*, nebkhas start to form by accumulation of wind-blown sand. As shown by field observations, branched *R. stricta* individuals had canopy diameters greater than about 0.25 m, and could be subdivided into infertile individuals (i.e. juveniles) and reproductive individuals (i.e. adults). The threshold diameter between branched juveniles and adults was about 0.50 m, as commonly observed in the field. Adult *R. stricta* shrubs can reach more than 4 m diameter, are auto-allelopathic, and are often one of the highest seed producing species within the community they establish [42], [43]. Seeds of *R. stricta* are rather large (about 2 mm wide and 6 mm long), and reside in follicles suspended from the parent plants; these follicles are roughly 4 mm wide and 50 to 100 mm long. Follicles normally open and release their seeds in the hot and dry conditions of late spring (i.e. April, May or June). The mechanisms through which *R. stricta* seeds disperse have not yet been uncovered, but large seeds are generally thought to be dispersed by vertebrates or water [44]. After wet season rains seeds may germinate, then become established (pre-reproductive) juveniles if they survive their first summer. Since our field data were recorded in fall 2010, and because (most likely) no rain had fallen since spring 2010, the majority of the unbranched individuals observed in the field had endured at least one summer, and were therefore considered established, juvenile individuals [44]. Most of these unbranched individuals were taller than 0.10 m; smaller ones probably died in summer conditions.

### Data collection

On 9 and 12 December 2010, we positioned a rectangular marker with known dimensions (2.9 m×1.9 m) in the study site, leveled it, and aligned it along the cardinal directions. We then mounted and gyro-stabilized a digital camera (Ricoh GX200) underneath a tethered helikite (a combination of a helium balloon and a kite which results in an aircraft that exploits both helium and wind for its lift); we used this helikite-camera combination to shoot aerial pictures of the entire study site. The helikite was guided along parallel linear tracks at low altitude (i.e. less than 10 m) to ensure a resolution of at least 100 pixels per m on each picture, while the camera was automatically triggered every five seconds to guarantee partial overlap between consecutive pictures. Three people were needed to properly direct the helikite along the parallel tracks: a controller controlling the tethered helikite, and two guides standing at opposite ends of a linear track. The guides had to give signals to the controller so that the helikite was always in between the guides (and thus on track). When a track was completely photographed, the two guides stepped towards the ends of the next parallel track to be photographed (5 m further). In this way, 995 study site pictures were taken (all in the afternoon), and were assembled into a topographic model of the study site using photogrammetric software (PhotoScan Pro). From the topographic model, an orthoimage was derived using the same software. The orthoimage resolution was set to 100 pixels per m (the scale was determined from the rectangular marker visible in the topographic model). The orthoimage was then manually divided into vegetation and bare soil classes with Photoshop CS5. *R. stricta* individuals were easily recognizable: branched ones from their large size compared to the image resolution, and unbranched ones from the clearly defined, long, linear shadows they produced. For all isolated vegetation patches we identified the centroid coordinates and surface area using MATLAB's Image Processing Toolbox (MATLAB R2011a). Vegetation patch diameters (Ø) were calculated from circles with areas set equal to those in the observed vegetation patches. Patches were subdivided into unbranched individuals (Ø<0.25 m), branched juveniles (0.25<Ø ≤ 0.50 m), small adults (0.50<Ø ≤ 1.00 m), medium adults (1.00<Ø ≤ 2.00 m) and large adults (Ø>2.00 m). We assumed that all patches with diameters larger than 0.50 m (i.e. all patches in the last three size classes) were reproductive adults. Unless stunted growth occurs, shrubs typically become larger with age; size and age are usually correlated shrub variables [45]. Since no visual signs of stunted growth were noticed on the field, we assumed that each of the size classes described above represents a distinct cohort (group of individuals with similar age), and that size classes with larger vegetation patches are older cohorts than size classes with smaller vegetation patches.

### Framework of hypotheses and associated patterns

**Figure 1:**
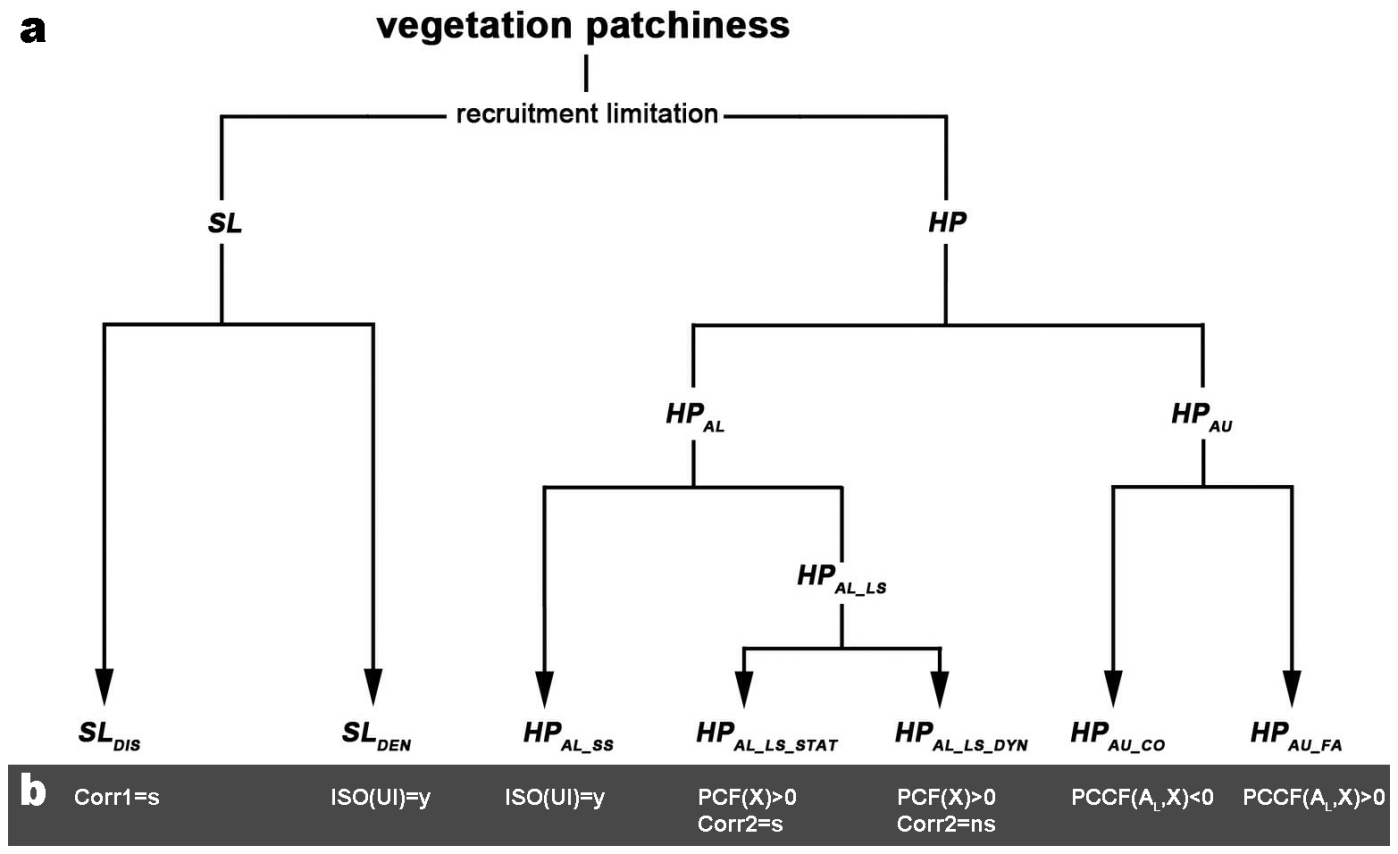
Schematic overview of the proposed hypotheses on *R. Stricta* recruitment dynamics, organized in a framework (subfigure a), together with their expected vegetation pattern characteristics (subfigure b). SL stands for seed limitation, HP for habitat patchiness, SL_DEN_ for density SL, SL_DIS_ for distance SL, HP_AL_ for allogenic HP, HP_AL_SS_ for small-scale allogenic HP, HP_AL_LS_ for large-scale allogenic HP, HP_AL_LS_STAT_ for static large-scale allogenic HP, HP_AL_LS_DYN_ for dynamic large-scale allogenic HP, HP_AU_ for autogenic HP, HP_AU_CO_ for competition, and HP_AU_FA_ for facilitation. ISO(UI) = y signifies that unbranched individuals occur isolated, PCF(X)>0 denotes that the evaluated PCF lies significantly (*p*<0.01) above the corresponding null model envelope (X representing the cohort under evaluation). PCCF(A_L_,X)<0 and PCCF(A_L_,X)>0 respectively indicate that the evaluated PCCF lies significantly (*p*<0.01) under and above the corresponding null model envelope (X and A_L_ respectively standing for a cohort other than the large adults, and the large adult cohort, between which PCCFs are being calculated). Corr1 = s denotes whether adult FVC spatially correlates significantly (*p*<0.05) with unbranched individual density. Corr2 = s or Corr2 = ns respectively mark whether the spatial correlations between densities of different cohorts are significant (*p*<0.05) or not.

We proposed a framework of hypotheses (Figure 1a) to represent the range of mechanisms possibly underlying the observed vegetation pattern. Each mechanism at the end of the framework's decision tree is associated with expected vegetation pattern characteristics (Figure 1b; see next section for definitions of pattern characteristics), which can be compared to the characteristics of the observed pattern, leading to acceptance or rejection of each hypothesized process. Pattern comparison was carried out with spatial statistical techniques described in the next section.

The proposed framework assumes that recruitment limitation underlies the observed vegetation patchiness in *R. stricta* nebkha fields, and that this limitation is either due to seed limitation (SL) or habitat patchiness (HP).

SL can moreover be spatially limited (i.e. distance SL) and density limited (i.e. density SL). Distance SL occurs when seeds cannot potentially reach all landscape locations as the seeds are restricted to parent plant surroundings (i.e. seeds stay within seed shadows). Offspring recruitment will consequently also be confined to parent plant surroundings under distance SL. Even when seeds can potentially reach all landscape locations, low seed densities might still be responsible for low vegetation cover. More specifically, density SL would lead to a distribution of isolated recruits (i.e. recruits of which the canopies do not touch, and are therefore surrounded by barren soil).

HP was considered the second possible cause of recruitment limitation (besides SL), and was either assumed underlain by allogenic (i.e. vegetation-independent) or autogenic (i.e. vegetation-dependent) drivers. Allogenic drivers can act at different spatial scales, possibly leading to distinctive recruitment patterns. For example, small-scale habitat patches that favored offspring recruitment might have been created by small-scale topographic depressions (0.1 m to 1 m scale) where soil water concentrated after rain. In contrast, recruitment might have been suppressed at a larger scale, for instance by background heterogeneity within the cemented floor, or due to rapidly deposited sand sheets (10 m to 100 m scales) which suppressed seedling emergence and killed seedlings by burial. These large-scale suppression processes thus might have been promoting large-scale allogenic HP (by top-down control), leading to large-scale clustering of offspring recruitment. In this respect, small-scale and large-scale were defined with reference to the shrub size scales defined for this study. *R. stricta* shrubs can reach up to 5 m diameter. Therefore, in the framework, allogenic processes responsible for habitat patches smaller than 5 m were considered small-scale, while those creating habitat patches larger than 5 m were assumed large-scale. Small-scale allogenic HP is supposed to produce isolated recruits, or small clusters (< 5 m) of recruits (when seeds are plentiful), while large-scale allogenic HP is supposed to generate large-scale clusters of recruits (> 5 m). Depending on the specific processes causing the large-scale HP, habitat patches might have been immobile during the nebkha pattern development process (resulting in static large-scale HP), for instance, due to large-scale heterogeneity inside the cemented floor. Suitability for recruitment would spatially, but not temporally, vary under static large scale allogenic HP, hereby forcing different cohorts of offspring recruitment in identical habitat patches, which in turn would create highly overlapping cohort clusters. In contrast, other large-scale allogenic processes may temporally vary, which might result in dynamic large-scale allogenic HP. For example, moving sand sheets and dunes might suppress seedling emergence and establishment (at different locations at different times). Hereby possibly creating separated spatial distributions of cohort vegetation patches.

In our framework, HP governed by autogenic processes (Figure 1a) was subdivided into competition and facilitation. We expected that competition would lead to recruitment suppression (and possibly also to suppression of other cohort shrubs) near large adults, either because of water and nutrient extraction by horizontally extended roots, or by allelopathy [46], [47]; auto-allelopathic effects have been observed with *R. stricta* [48]. Conversely, we expected elevated recruitment success (or an elevated density of shrubs of other cohorts) near facilitating large adults, either because of lateral water diffusion from large adults due to higher infiltration rates on nebkhas, a decrease in sand abrasion, providing shade, or increased nutrients around nebkhas from litter and fine soil accumulation.

### Hypothesis assessment through pattern analysis

**Figure 2:**
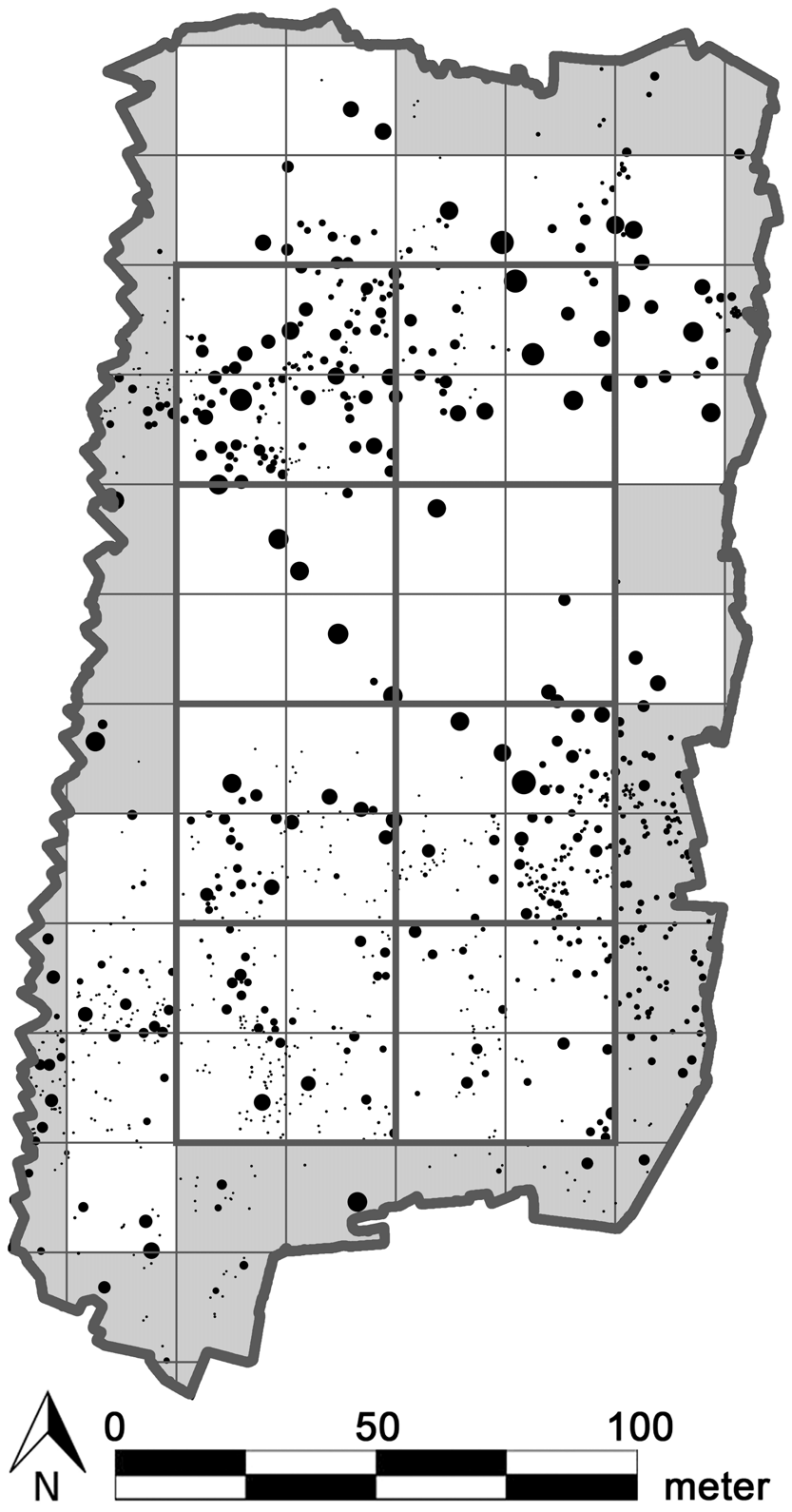
Study area with *R. stricta* patches displayed as discs. To make all unbranched individuals visible in this figure, they were enlarged to discs of 0.25(i.e. all branched individuals) are displayed proportional to their actual surface area. A grid with square plots with sides five times the diameter of the largest observed nebkha (5×4.2 m = 21 m) is overlain over the site. Only cells with white background completely fall inside the study site. Gray plots were omitted from the calculations. A second grid with cell sides ten times the diameter of the largest observed nebkha (10×4.2 m = 42 m) is also overlain. The sides of these grid cells (8 in total) are depicted in bold.

Presence of isolated unbranched individuals was assessed through visual inspection of the orthoimage of the study site. Isolated unbranched individuals are signs of density SL or small-scale allogenic HP. However, to distinguish density SL from small-scale allogenic HP, seed-addition experiments are needed, as this distinction cannot be made from mere pattern analysis alone. Since we presumed that seed outputs of reproductive plants (i.e. adults) are mainly determined by their canopy size [49], we tested for distance SL by evaluating the correlation between the fractional vegetation cover (FVC) of adults and the densities of unbranched individuals (the youngest cohort). Both adult FVC and unbranched individual density were measured in square plots (Figure 2). As the size of the spatial distribution of seeds, as dispersed from a single plant, can vary [50], two sizes of square plots were used: with sides of five times and ten times the diameter of the largest observed vegetation patch (Figure 2). Distance SL was not rejected when one of the above-described correlation coefficients was significant (*p*<0.05).

**Figure 3:**
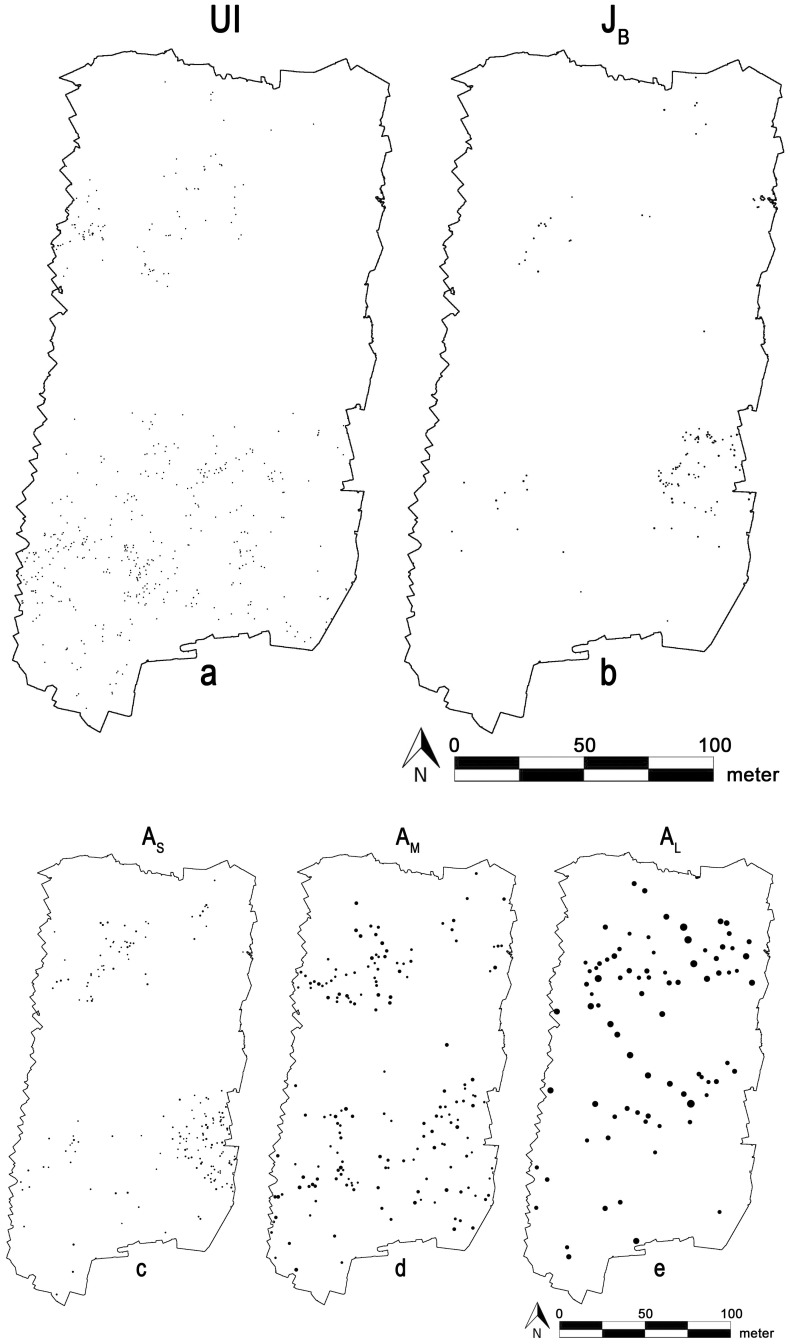
Five size classes of *R. stricta* patches (assumed as cohorts), separately depicted in the study site. Patterns correspond to (a) unbranched individuals (UI, N = 500), (b) branched juveniles (J_B_, N = 125), (c) small adults (A_S_, N = 178), (d) medium adults (A_M_, N = 171), and (e) large adults (A_L_, N = 82). To make all unbranched individuals visible, they were enlarged to discs of 0.25 m diameter, regardless of their actual size. Vegetation patches of the other cohorts are displayed proportional to their actual surface areas. Unbranched individuals (a) and branched juveniles (b) are depicted at a larger scale (together with a larger scale bar) to better visualize the cohorts with the smallest vegetation patches.

We evaluated large-scale allogenic HP by testing whether cohorts grouped into large-scale clusters (>10 m diameter). We applied the pair correlation function (PCF) and an associated null model (for both, see next section) to each observed cohort pattern. Large-scale allogenic HP was subdivided in a static and dynamic type (Figure 1a). The static type refers to immobile large-scale allogenic processes, which because of their immobility, force different cohorts to appear in highly overlapping large-scale areas. In contrast, the dynamic type refers to mobile large-scale allogenic processes, from which distinct cohorts are expected to appear in poorly overlapping large-scale areas (due to the mobility of the underlying large-scale processes). To distinguish between both, we again used the square plots depicted in Figure 2 (but now only the small ones), and tested whether the vegetation patch densities inside the square plots correlated between cohorts (Figure 3). The fewer significant spatial correlations between cohort patch densities, the stronger the evidence that large-scale allogenic HP was dynamic rather than static, as this would suggest a weakening of spatial overlap between large-scale clusters of different cohorts.

Because we assumed that larger vegetation patches produced stronger autogenic effects than smaller vegetation patches, we considered large adults (Ø>2 m) as the generator of autogenic effects. To detect autogenic HP, we employed the pair cross-correlation function (PCCF) together with an associated, null model (for both, see next section), and tested whether the spatial distribution of each cohort (other than the large adults) spatially correlated with that of the vegetation imposing autogenic influence (i.e., the large adults), either negatively (suggesting competition) or positively (indicating facilitation).

Most of the hypotheses in the framework (Figure 1a) can co-occur, although the static and dynamic type of large-scale allogenic HP cannot go together. Vegetation pattern characteristics expected to arise from each proposed hypothesis were listed under the leaves of the hypothesis tree (Figure 1b).

### The PCF, PCCF, and associated null models

We used the PCF to assess vegetation patch clustering inside one pattern (i.e. inside each cohort), and the PCCF to detect competition and facilitation processes between patches of large adults and other cohorts. The original PCF as described by Diggle [51] compares the frequency distribution of point pair distances between a univariate point pattern under study and an equally dense, completely spatially random point pattern. The PCCF is the bivariate PCF form, and only considers distances between points of two different patterns (in our case: two different cohorts). Both PCF and PCCF are second-order spatial statistics as they are based on distances between point pairs, in contrast to first-order spatial statistics which are based on absolute point positions, such as point density [52]. However, we analyzed patches instead of points, and therefore used the PCF modification as proposed by Nuske [53] where abscissa values represent shortest distances between patch-edges instead of distances between patch-centroids. We proposed in this study a similar modification for PCCFs. To correctly interpret the PCF and PCCF results in this study, we compared the calculated PCFs and PCCFs with PCF and PCCF envelopes which comprise 499 patterns resulting from Monte Carlo simulations of appropriate null models (explained below). We proposed and used a univariate and a bivariate null model. R code was written to create the null models (see Appendix S1) and to modify existing R code of default PCF and PCCF functions from the spatstat package [54]. The Monte Carlo simulations were run in R (version 3.0.2).

The univariate null model was designed to detect large-scale vegetation patch clusters in cohorts. It randomized, for a given cohort, observed cohort patch centroid coordinates according to the homogeneous Poisson process (which resulted in patterns with spatially constant density). However, the randomized vegetation patches of the analyzed cohort were not only not allowed to overlap with each other, but also to not overlap with vegetation patches of older cohorts (which were not randomized) [55]. Centroids of patches to be randomized were (one at a time, from largest to smallest) placed randomly inside the study site. To assure non-overlap between vegetation patches, randomization attempts were, when necessary, repeated until patches did no longer overlap. The bivariate null model was designed to detect repulsion (e.g. competition) and attraction (e.g. facilitation) between large adults and vegetation patches of other cohorts. This null model spatially randomized a given (non-large adult) cohort according to a heterogeneous Poisson process which preserved the observed spatially variable vegetation patch density (again with the restriction of non-overlap between the randomized and previously established vegetation). In this way, possible large-scale cohort clusters (resulting from large-scale allogenic HP) were preserved into the bivariate null model simulations, which is necessary if we were to detect attraction (facilitation) or repulsion (competition) processes between large adults and other cohort vegetation. Indeed, mere homogeneous randomization could obscure such interaction processes [55], [56] when superimposed on large-scale allogenic HP. To define the spatial density function of this heterogeneous Poisson process, we employed an isotropic 5 m standard deviation 2-dimensional Gaussian kernel. We found this kernel size low enough to reveal present large-scale cohort clusters (local patches of high vegetation patch density), while not too low to include possible repulsion or attraction processes into the null model simulations (which, as explained before, would leave these interaction processes undetectable).

For each null model, we produced two sets of Monte Carlo patterns; a first one to detect distance intervals over which PCFs and PCCFs of studied patterns fall outside PCF and PCCF-envelopes, and a second one to test the significance of each deviating distance interval by goodness-of-fit (GoF) tests. GoF tests are necessary as the significance level of envelope departures can only be assured for single deviating distances with PCFs and PCCFs, not for deviating distance intervals. For each deviating distance interval, significance testing was then performed by firstly converting all included PCF and PCCF-values to a univariate GoF test statistic *u* (see Loosmore [57] for the exact test-statistic used), for the observed pattern *u_o_* and for each simulated pattern (*u_i_*, i ***?*** [1, …, 499]). When *u_o_* belonged to the five highest or five lowest values in the set of all 500 values (499 *u_i_* and 1 *u_o_*), the null model was rejected for the distance interval under study with type I error rate 0.01 [58]. The deviation strength of a deviating distance interval is proportional to the PCF and PCCF envelope's distance to the null line, and is inversely proportional to the PCF and PCCF envelope's width.

## Results

### Non-spatial pattern characteristics

We observed a total of 1056 (370 ha^−1^) *R. stricta* patches, of which 500 were UI, 125 J_B_, 178 A_S_, 171 A_M_, and 82 A_L_ (Figure 3). *R. stricta* total and unbranched individual FVC was 3.01% and 0.01%, respectively. Visual inspection of the study site's orthoimage revealed isolated occurrences of unbranched individuals, from which we derived suggestive evidence for density SL or small-scale allogenic HP.

**Figure 4:**
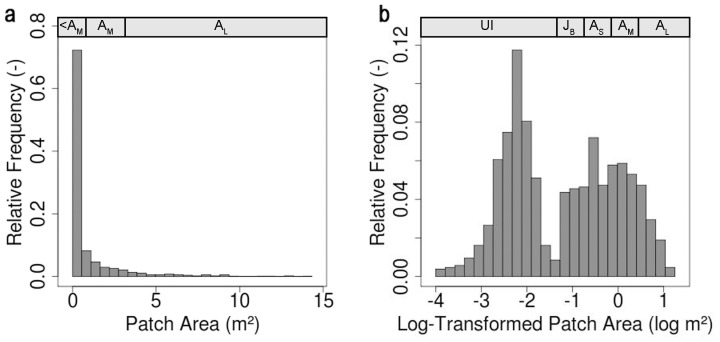
Histograms with 25 bins of *R. stricta* patch (a) surface areas and (b) log-transformed surface areas. Size classes (assumed cohorts) of unbranched individuals (UI), branched juveniles (J_B_), small adults (A_S_), medium adults (A_M_), and large adults (A_L_) are depicted inside the histograms, where possible. However, due to lack of space in the left histogram, size classes smaller than A_M_ are joined together (denoted as<A_M_).

The largest diameter of all observed *R. stricta* patches was 4.2 m, and therefore the square plots in Figure 2 have sides of 21 m (5×4.2 m) and 42 m (2×21 m; bold grid cells). The frequency distribution of *R. stricta* patch areas is right skewed and unimodal (Figure 4a). However, the frequency distribution of log-transformed patch areas is clearly bimodal (Figure 4b). Interestingly, the abscissa value corresponding to the local minimum between both peaks in Figure 4b exactly matches the border between the unbranched and branched size classes.

### Spatial pattern characteristics

**Figure 5:**
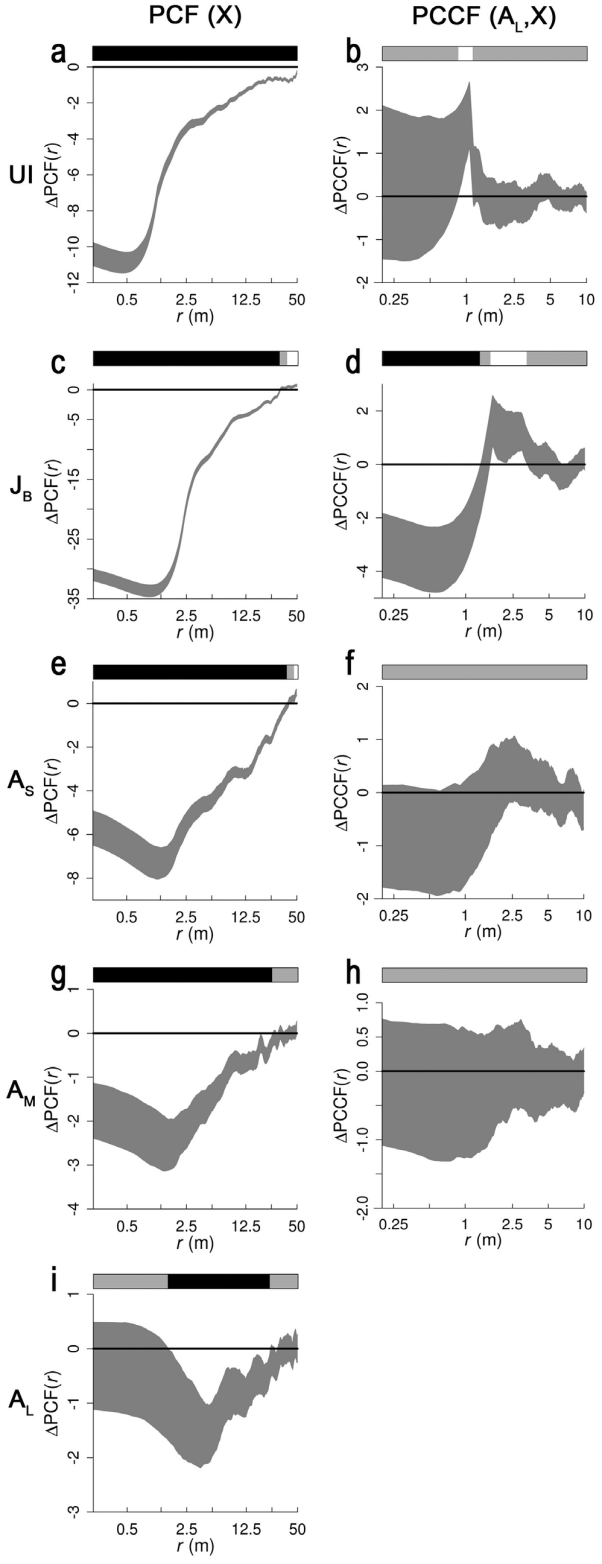
Spatial second-order summary statistics of five size classes, alone (univariate), and related to the large adult *R. stricta* pattern (bivariate). The left panels display the PCFs of the five size classes, while the right panels list the PCCFs between the large adult size class and four other size classes [unbranched individuals (UI), branched juveniles (J_B_), small adults (A_S_), and medium adults (A_M_)]. X represents the size class under analysis (either UI, J_B_, A_S_, A_M_ or A_L_). Each PCF envelope is comprised of the minimal and maximal values in the PCF-set resulting from 499 simulated univariate null model patterns, while each PCCF envelope is comprised of the minimal and maximal values in the PCCF-set resulting from 499 simulated bivariate null model patterns (see Methods for null model descriptions). PCFs and PCCFs of observed patterns are subtracted from their associated null model envelopes. In this way only differences between observed PCFs and PCCFs and their respective envelopes are shown, as indicated by ΔPCF(*r*) and ΔPCCF(*r*), where *r* represents pair distances expressed in meters. Envelopes that fall completely above or beneath the null line, for a range of pair distances, therefore indicate deviations from randomness at those scales. Black and white intervals in bars above the graphs, respectively pinpoint significant positive deviations (indicating clustering for PCFs, and bivariate clustering for PCCFs) and significant negative diversions (indicating regularity for PCFs, and bivariate regularity for PCCFs) from envelopes, while gray intervals indicate scale intervals where the null model is not rejected according to GoF tests (*p* = 0.01).

No evidence was found for distance SL (which was therefore rejected), since the FVC of vegetation assumed reproductive (i.e. adults) did not correlate with the unbranched individual density (*p*>0.05), neither when measured in 21 m nor in 42 m square plots (Figure 2). The spatial distribution of *R. stricta* cohorts however deviates strongly from randomness (Figures 5a,c,e,g,i), which implies that in each cohort, the vegetation patches were significantly clustered (*p*<0.01). These cluster diameters exceeded 10 m, as the envelopes fall underneath the null line up to more than 10 m distances (Figures 5a,c,e,g,i). However, with increasing cohort age, the cluster diameter tended to decrease: the average cluster diameter was larger than 50 m for unbranched individuals, while it was about 22 m for large adults. The clustering strength (measured as the envelope's null line deviation, relative to the envelope's width) also progressively decreased with increased size class (Figures 5a,c,e,g,i). We ascribed the large-scale clustering of *R. stricta* cohort patches to large-scale allogenic HP. Vegetation patch densities, as measured in 21 m square plots (Figure 2), between most (of all 10 possible) cohort pairs were uncorrelated (*p*>0.05; Table 1). However, between medium adults and small adults, between medium adults and branched juveniles, between medium adults and unbranched individuals, and between small adults and juveniles, vegetation patch densities showed a significant correlation (*p*<0.05). As most cohort pairs did not have spatially correlating vegetation patch densities, we concluded that the earlier inferred large-scale allogenic HP was dynamic in space.

**Table 1:** Correlation coefficients regarding *R. stricta* densities in 21 m square plots between different *R. stricta* size classes (assumed different cohorts).

	UI	J_B_	A_S_	A_M_
UI	/	/	/	/
J_B_	0.06	/	/	/
A_S_	0.12	0.76^***^	/	/
A_M_	0.34^*^	0.48^*^	0.65^***^	/
A_L_	0.20	0.05	0.09	0.15

Included size classes are: unbranched individuals (UI; Ø<0.25 m), branched juveniles (J_B_; 0.25 m<Ø<0.50 m), small adults (A_S_; 0.50 m<Ø<1.00 m), medium adults (A_M_; 1.00 m<Ø<2.00 m) and large adults (A_L_; Ø>2.00 m). Significant correlations are flagged with 1, 2 or 3 asterisks, representing *p*-values between 5 10^−2^ and 10^−3^, between 10^−3^ and 10^−4^, and below 10^−4^, respectively. As correlation matrices are symmetric, only the lower triangular part is shown.

Unbranched individual densities significantly (*p*<0.01) decreased around 1 m from large adult edges (as measured with the PCCF), from which we assumed competition, but this was only observed across a tiny scale range (Figure 5a). Strong indications were found that large adults competed significantly (*p*<0.01) with branched juveniles, between 1.5 and 3.25 m from large adult edges. However, large adults seemed to additionally have facilitated branched juveniles very close to their edges (i.e. less than 1 m) (Figure 5d). Both forms of autogenic HP (i.e. competition and facilitation) were therefore assumed present in our study site. All proposed *a priori* hypotheses on nebkha recruitment are, together with their evaluation, summarized in Table 2.

**Table 2:** Hypotheses assessment summary.

Hypotheses:	SL_DIS_	SL_DEN_	HP_AL_SS_	HP_AL_LS_STAT_	HP_AL_LS_DYN_	HP_AU_FA_	HP_AU_CO_
Supported:	n	y	y	n	y	y	y

The hypotheses at the end of the framework's decision tree (Figure 1a) are listed in the upper row. The bottom row states the decision for each hypothesis. Rejected hypotheses are indicated by “n”, supported ones by “y”. SL stands for seed limitation, HP for habitat patchiness, SL_DEN_ for density SL, SL_DIS_ for distance SL, HP_AL_ for allogenic HP, HP_AL_SS_ for small-scale allogenic HP, HP_AL_LS_ for large-scale allogenic HP, HP_AL_LS_STAT_ for static large-scale allogenic HP, HP_AL_LS_DYN_ for dynamic large-scale allogenic HP, HP_AU_ for autogenic HP, HP_AU_CO_ for competition, and HP_AU_FA_ signifies facilitation.

## Discussion

The low total (3.01%) and unbranched individual (0.01%) fractional vegetation cover fits the assumption that our study site underwent severe recruitment limitation. The isolated occurrence of unbranched individuals (and vegetation patches of other cohorts) moreover indicates density SL or small-scale allogenic HP. Most likely density SL and small-scale allogenic HP co-occurred [23], but only one of these two usually dominates [59]. Although *R. stricta* produces many seeds, these are often not found in soil seed banks of communities with *R. stricta* vegetation [42], [43], probably because they quickly decay or are rapidly preyed upon. This suggests that our study site lacked *R. stricta* seeds. However, density SL cannot be solely inferred from lack of seeds. To deduce density SL for certain, artificial seed addition would need to yield more vegetation cover (initially in the form of seedlings). The relative importance between density SL and small-scale allogenic HP should therefore be determined with additional experiments in which various seed densities are added to plots (with similar pre-existing unbranched individual densities), and where, in the next growing season, the seedling densities are counted and compared to each other [60], [61]. If many additional seedlings were to arise from the added seeds, density SL would be shown to be more important than small-scale allogenic HP (and *vice versa*).

We propose three drivers of dynamic large-scale allogenic HP, meaning three processes which might cause a combination of within-cohort clustering of *R. stricta* vegetation patches, and the spatial segregation between cohort clusters. The first two proposed processes involve wind-blown sand movement, and the third one human disturbance. Large-scale sand bodies, such as sand sheets and dunes are typically highly dynamic in space and time [6], [62]. Seeds might therefore be buried too deeply to germinate, while seedlings may be killed by denudation [63], [64] or sand burial [65]. Seedling emergence in desert shrubs has been proven highly sensitive to minor changes in seed burial depth. Experiments with different desert shrub species indeed show that starting from the soil surface, the chance of seedling emergence from seeds initially increases (since most seeds do not germinate at the soil surface) toward an optimal burial depth (often situated between 10 and 20 mm). When the seeds are buried deeper, chances of seedling emergence decline drastically [25], [66], [67], [68], [69]. Desert shrub seedlings also respond to burial depth, as they usually survive (and sometimes benefit from) partial sand burial; although most seedlings will die after complete burial [2], [70]. As a second driver, aeolian processes causing dynamic topographic variation, might create spatially non-uniform distributions in plant-available soil water [71]. The third driver that may lead to the dynamic clustering of cohort vegetation patches is, we argue, human disturbance induced by trails of camels and motor vehicles. Indeed, the trampling and soil compaction associated with these trails may hamper vegetation establishment even after the pathways have been abandoned for years [72]. When such trails slowly reroute following evolving vegetation configurations, they might contribute to dynamic large-scale allogenic HP.

Although the inferred large-scale allogenic HP was considered dynamic, not all ten cohort pair combinations were spatially uncorrelated. Four cohort pairs had partially overlapping spatial configurations (i.e. patch densities that correlated in space; Table 1), suggesting that some cohorts established in highly overlapping large-scale habitat patches. We postulated that this was caused by slowly moving habitat patches (slow relative to time intervals between establishment dates of consecutive cohorts). Indeed, gradual large-scale habitat patch movements would give consecutive cohorts a higher chance than non-consecutive cohorts in establishing themselves in partially overlapping habitat patches. The two most significant (*p*<10^−4^) and strongest cohort density correlations were indeed those between consecutive cohorts.

The commonly cited stress gradient hypothesis (SGH) places autogenic processes (i.e. competition and facilitation) in a theoretical framework. The SGH generally states that, depending on the level of abiotic environmental stress, either competition or facilitation will prevail. Under this theory, competition is expected to dominate under benign conditions, and facilitation under harsh abiotic ones [73], [74]. As our hyper-arid study site can be considered as highly stressed (water and nutrients are scarce), facilitation should dominate according to the SGH. We observed strongly suggestive evidence of both competition and facilitation in our study site: competition between large adults and unbranched individuals (Figure 5b), and both competition and facilitation at different spatial scales between large adults and branched juveniles (Figure 5d). Our analysis however suggests that the competition between large adults and the youngest cohort (i.e. the unbranched individuals) was almost nil (Figure 5b). The effect of the large adults on the second youngest cohort (i.e. the branched juveniles) was more prominent: the density of branched juveniles was significantly (and strongly) higher up to 1 m from large adult edges, while being significantly (but moderately) lower between 1.5 m and 3.25 m away (Figure 5d). These results suggest that facilitation was the strongest present autogenic process, which fits the SGH. Remarkable is that autogenic processes arising from large adults appear to have poorly affected the youngest cohort (Figure 5b), clearly affected the second youngest cohort (Figure 5d), and (again) did not affect the third (Figure 5f) and fourth (Figure 5h) youngest cohorts. The former two observations may indicate that unbranched individuals initially established equally well everywhere, but that they had lower mortality rates near large adults, thereby leading to higher branched juvenile densities near large adults. Such a decline in recruit mortality near large shrubs might be caused by a reduction of sand drift (and thus sand abrasion) in sandy environments [63], [75]. It might also be that unbranched individuals did not yet encountered high winds (and thus high sand abrasion) during their lifetime, thereby making potential large adult facilitation on unbranched individuals invisible. Large adults could have provided shade to surrounding vegetation, and thereby as well possibly decreased mortality of nearby small vegetation patches [76]. Cohorts older than juveniles did not show increased densities near large adults which might suggest that growing branched juveniles can merge with large adults, as indeed the inferred facilitation zone around large adults is limited (< 1 m) with respect to the sizes of small and medium adults (Ø>0.5 m). Merging shrubs may die due to competition for space. The inferred combined nearby facilitation and distant competition between large adults and branched juveniles (Figure 5d) might have been a superimposed short-ranging facilitation and long-ranging competition (or a so-called scale-dependent feedback [77]).

To bring more clarity to the exact causes and effects of competition and facilitation processes within *R. stricta* nebkha patterns, future studies might include soil analyses, seedling addition experiments and follow-up remotely sensed information. Analyses on soil samples at varying distances from large nebkhas might ascertain the sources of the inferred competition and facilitation processes. Mortality rates of seedlings in function of distance to large nebkhas (through experiment or follow-up imagery), and observation of shrub merging (through follow-up imagery), may confirm the results of this study.

Degrees of clustering declined within cohorts age, since deviations between the PCF envelope and the null line (as measured relative to the envelope width) are smaller with older cohorts (see Figure 5 c,e,g,i). A weakened clustering with cohort age is often reported in studies of arid environments [13], [14], [78]. However, erroneous methodology may be the underlying cause. Indeed, when vegetation patches overlapped during null model randomizations, observed patterns would appear too regularly distributed against null model simulations (when vegetation patches were initially observed as spatially isolated). As such bias increases with vegetation patch size, clustering degrees of older cohorts (with larger vegetation patches) might incorrectly appear to decrease [53], [79]. However, the null models used in this study did not allow patch overlap during randomization. Hence erroneous methodology did not underlie the observed clustering decrease with cohort age in our study site. Density-dependent mortality is often cited to weaken clustering, but even density-independent mortality might under certain conditions lead to the same result [78], as also the merging of neighboring individuals within cohorts.

Our study results strongly indicated that allogenic processes (more specifically dynamic large-scale allogenic HP) had much more impact on the observed overall vegetation pattern, than autogenic processes (i.e. competition and facilitation). Presumed autogenic effects had to be rather weak (considering the relatively minor envelope deviations as compared to the envelope widths) and completely disappeared between large adults and cohorts older than juveniles. According to our analyses, inferred autogenic processes only worked on a small fraction of the study site area. These reasons most likely imply that autogenic processes were not main drivers of recruitment limitation in our study site. The inferred low autogenic impact in our study site contrasts with many other field sites in arid regions, where vegetation patterns are primarily shaped by autogenic processes rather than by allogenic ones [31]. The difference in autogenic impact might be caused by the hyper-aridity of the study site for which the classic SGH may not be valid [80], or it may originate from the abiotic stress created by aeolian (i.e. allogenic) forces on sandy top soil (e.g. burial and abrasion by sand).

We conclude that both SL and HP most likely caused the observed *R. stricta* spatial pattern. Seed dispersal was probably density limited (i.e. density SL), but not spatially confined due to seed shadows (i.e. distance SL). Strong suggestive evidence indicates that the previously inferred HP was driven more strongly by allogenic than by autogenic processes, and that these allogenic drivers forced *R. stricta* offspring into large-scale clusters. Depending on the year of recruitment, offspring clusters might have emerged at different locations. Each studied cohort was clustered, but less so with increasing age, possibly due to the coalescence of neighboring shrubs.

Overgrazed, hyper-arid regions are often prone to land degradation and soil erosion, especially under climate change [36]. Under such conditions, introduction of unpalatable nebkha host plants (e.g. *R. stricta*), for example by planting of cuttings, might be a key solution to restrain desert expansion and sand drifts. As a first step towards practical land management, the present study aimed to perform basic research about the main drivers underlying the natural regeneration processes of unpalatable nebkha host plant species, under above described conditions. Our findings suggest that in a seemingly homogeneous hyper-arid environment, dynamic large-scale heterogeneity might render large-scale areas less suitable for plantation of nebkha host plant cuttings. However, to be of practical use in future land management, further (more experiment-based) research is essential to help uncover the specific physical processes underlying the presumable dynamic large-scale habitat patchiness indicated in this study.

## Supporting Information

Appendix S1 **R code of null models.**
(DOCX)
