# Supplementary material for: Use of Spatial Analysis to Test Hypotheses on Plant Recruitment in a Hyper-Arid Ecosystem
Source: PLoS One. 2014 Mar 10;9(3):e91184. doi: 10.1371/journal.pone.0091184 (PMC3948780; doi:10.1371/journal.pone.0091184)
Supplement: Appendix S1 — R code of null models. (DOCX) [file pone.0091184.s001.docx]

**Appendix S1: R code of null models**

**nullmodel <- function (nlargefixed,vpp,window=W,giveup=100000,usedensitymap=F){**

#This R function can generate simulated vegetation patterns according to the null models described in this manuscript. Therefore, the observed vegetation pattern will firstly be split in a pattern of vegetation patches that not needs to get randomized (here defined as the ‘fixed pattern’), and in a pattern of vegetation patches that needs to get randomized (here defined as the ‘unfixed pattern’). The fixed and the unfixed pattern are created by a threshold radius, so that the fixed pattern contains all patches of the observed vegetation pattern with radii larger than this threshold radius, and so that the unfixed pattern contains all remaining patches (i.e. the patches not present in the fixed pattern). By adjusting the function arguments, all null models as described in the manuscript can be attained. These function arguments are: 'nlargefixed', an integer, representing the number of patches in the fixed pattern (and thus indirectly representing the threshold radius); 'vpp', a marked spatial pattern (a spatstat object), representing the observed vegetation patch pattern with as marks the radii of the observed vegetation patches; 'window', an owin (a spatstat object), representing the study site border (or the study site window); 'giveup', an integer, representing the maximum possible number of attempts to place a given vegetation patch centroid inside the study site window, without overlapping other vegetation patches; 'usedensitymap', a boolean which denotes whether the spatial density of the unfixed pattern should be retained during the randomization process (usedensitymap=T) or not (usedensitymap=F).

**require(spatstat)**

#the package spatstat is required to use this function

**radii = marks(vpp)**

#the vector ‘radii’ is defined; it contains all vegetation patch radii of 'vpp'

**n <- length(radii)**

#'n' equals the number of vegetation patches in 'vpp'

**radii <- sort(radii,decreasing=TRUE)**

#’vpp’ vegetation patch radii are sorted from largest to smallest

**X=vpp[vpp$marks>=(sort(vpp$marks)[vpp$n+1-nlargefixed])]**

#'X' represents the fixed pattern

**radii <- radii[(nlargefixed+1):n]**

#'radii' is redefined, now only containing the radii of the unfixed pattern

**n <- n-nlargefixed**

#'n' is redefined, now representing the number of patches in the unfixed pattern

**r <- radii[1]**

#'r' is defined as the first element of 'radii' (being the largest patch radius of the unfixed pattern)

**X$window <- window**

#the study site window is attached to the unfixed pattern.

**for(i in 1:n){**

#In this for-loop, attempts will be made to randomize patches of the unfixed pattern without overlap (one be one, and from largest to smallest radius). Once a patch of the unfixed pattern is successfully randomized without overlap, it will become part of the fixed pattern ‘X’.

**K <- 0**

#‘K’ denotes the number of randomization attempts (of the current patch to be randomized)

**r <- radii[i]**

#'r' is redefined as the i-th element of 'radii'

**W <- erosion(window,r)**

#since patch centroids are randomized, the borders of the study site need to shrink by a distance equal to the radius of the current (to be randomized) vegetation patch (resulting in ‘W’), as to guarantee that all randomized patches completely fall within the study site. However, not all of these randomizations are accepted, only those without overlap (see code below).

**repeat {**

**if(usedensitymap) {Y <- rpoint(1,W,f=densitymap)} else {Y <- runifpoint(1,W)}**

#a single point pattern is created by placing a point randomly inside the empty window ‘W’. If ‘usedensitymap==TRUE’, chances of a point being somewhere placed are spatially dependent, and proportional to the local patch density of the observed unfixed pattern. For the univariate null model, ‘usedensitymap’ needs to be set to ‘FALSE’, for the bivariate null model, ‘usedensitymap’ needs to be set to ‘TRUE’.

**dmatrix <- crossdist(X,Y)**

#’dmatrix’ is a matrix of all distances between the single point pattern Y (i.e. the present randomization attempt of a patch), and the fixed pattern ‘X’.

**if(all(dmatrix>= marks(X) + r)) break**

#accept the randomized patch if it does not overlap any patches of the (current) fixed pattern .‘X’

**K <- K+1**

**if(K%%1000 == 0) cat(K,"tries\n")**

**if(K >= giveup) stop(paste("Giving up after ",giveup," attempts ","to place ",i,"-th point ","with disc radius ",r,".\n",sep=""))**

#screen output.

**}**

**Y$window <- window**

**marks(Y) <- r**

#the mark (radius ‘r’) is attached to the accepted randomized patch centroid ‘Y’.

**X <- superimpose(X,Y)**

#add the randomized patch ‘Y’ to the fixed pattern ‘X’.

**}**

**X**

#’X’ now represents the final pattern, randomized according to the null model.

**}**
